# Development of 3D Printed Networks in Self-Healing Concrete

**DOI:** 10.3390/ma13061328

**Published:** 2020-03-14

**Authors:** Cristina De Nardi, Diane Gardner, Anthony Duncan Jefferson

**Affiliations:** Resilient Materials for Life (RM4L) Research Group, School of Engineering, Cardiff University, Wales CF243AA, UK; gardnerdr@cardiff.ac.uk (D.G.); jeffersonad@cardiff.ac.uk (A.D.J.)

**Keywords:** mini-vascular networks, self-healing, concrete, sodium silicate

## Abstract

This paper presents a new form of biomimetic cementitious material, which employs 3D-printed tetrahedral mini-vascular networks (MVNs) to store and deliver healing agents to damage sites within cementitious matrices. The MVNs are required to not only protect the healing agent for a sufficient period of time but also survive the mixing process, release the healing agent when the cementitious matrix is damaged, and have minimal impact on the physical and mechanical properties of the host cementitious matrix. A systematic study is described which fulfilled these design requirements and determined the most appropriate form and material for the MVNs. A subsequent series of experiments showed that MVNs filled with sodium silicate, embedded in concrete specimens, are able to respond effectively to damage, behave as a perfusable vascular system and thus act as healing agent reservoirs that are available for multiple damage-healing events. It was also proved that healing agents encapsulated within these MVNs can be transported to cracked zones in concrete elements under capillary driving action, and produce a recovery of strength, stiffness and fracture energy.

## 1. Introduction

Self-healing in cementitious materials has received significant interest over the last 2 decades. The potential of self-healing cementitious material systems is considerable since they negate the need for manual repairs and produce significant environmental and economical savings. Many of the healing systems developed use encapsulated healing-agents and/or additions to the cementitious matrix; which includes mineral admixtures [1,2,3], fibres [4,5] polymers [6,7] and/or nanoparticles [8]. Some of these have been added directly to concrete mixes in their natural form, whilst others have been encapsulated inside hollow pipettes/tubes or microcapsules.

In 1994, a research group at the University of Illinois [5,6,7,8] was one of the first to introduce the idea of autonomous healing in concrete using the active and passive-mode release of adhesive from glass tubes under cracking. Dry [9] tested a three-part methylmethacrylate (MMA) adhesive system; initially, the three adhesive parts were stored inside hollow glass tubes, but the tendency of these to break prematurely resulted in the use of hollow tubes coated with a brittle sealer. Results clearly showed that the MMA adhesive restored strength and made the specimens more flexible. Following the work by Dry, a number of other investigators explored cementitious material systems that used brittle material capsules [10,11,12,13].

Several research studies have focused on the best prophylactic agent, with a number of investigators acknowledging the need for a low viscosity liquid, which can reach damage zones easily and fill cracks of varying widths. Among the air-curing healing agents available, cyanoacrylates (CA) have been extensively studied due to their good rheological properties and ability to prevent crack re-opening thanks to their relatively high strength, adhesion and stiffness [14,15]. Silicons [16] and alkali-silica solutions [17], as well as epoxies [18] and polyurethanes [19], have been also tested in healing systems. Over the last decade, several studies have focused on the use of sodium silicate solution (SS), which reacts with calcium hydroxide (CH) to form a Calcium Silicate Hydrate (C-S-H) gel within cracks [20,21]. Pelletier et al. [22], highlighted that the presence of polyurethane capsules with a core of SS resulted in a 10% increase in flexural performance, when compared to the control samples. Recently, studies conducted by Formia et al. [23] have underlined the ability of SS to flow out from hollow cementitious tubes; samples tested in flexure 10 days after the first pre-loading stage showed an average strength and stiffness recovery index of 8% and 23% respectively. Moreover, when the time of healing was extended to three weeks, the average strength recovery index increased to 14%.

There are, nevertheless, several disadvantages to capsule and closed tube-based systems, as observed by Joseph et al. [24]. These authors showed that closed capillary tubes embedded in mortar samples were unable to release CA into cracks in sufficient quantity to achieve healing. A large amount of both CA and ink (the latter used as a control liquid) was found to have remained inside the tubes, indicating that tubes with closed ends have a higher capillary resistive force than the capillary attractive force associated with the crack.

In recent years, the use of vascular network channels, which mimic blood and nutrient circulation networks in biological systems, has been proposed by several researchers to overcome the weaknesses associated with hollow capsules. Minnebo et al. [25] developed a vascular network system that comprised a series of parallel tubes connected to a 3D printed distribution piece together with an external reservoir to supply the healing agent. It was noted that the presence of the 3D printed distribution piece had a negative impact on the mechanical behaviour of the samples in both the pre-peak and post-peak loading regime. Nevertheless, a recovery in mechanical properties was observed, and multiple healing cycles were achieved. Mimicking bone shape materials, Sangadji et al. [26] developed a porous network system similar to ‘spongious bone’; the healing mechanism was induced by the manual injection of epoxy resin. Test results from cylindrical and beam specimens showed a recovery in strength post-healing.

Recently, researchers at Cardiff University [27] have developed a method for creating 2D vascular networks using removal polyurethane tubes to create hollow interconnected channels within cementitious matrices and proven the viability of this healing system in a set of full-scale site trials [28,29]. However, despite these successes, vascular networks are not favoured by contractors because network forming operations are disruptive to the concrete manufacturing and casting process.

An alternative, more convenient, approach is herein proposed which uses Mini-Vascular Networks (MVNs) that can be added to wet concrete during the mixing process. The MVNs considered in this study are 3D printed tetrahedral units (named TETs) of interconnected hollow ligaments that have a characteristic dimension (i.e. maximum ligament length) approximately twice the size of the maximum aggregate particle size of the cementitious composite.

The aim of the work reported in this paper was to design an MVN and determine its effectiveness in a concrete healing system. The structural elements used in the study were prismatic concrete beams containing healing-agent filled 3D printed tetrahedral MVNs. A number of variables were considered in the study, including the (i) number of MVNs per unit volume of concrete; (ii) relative position of MVNs within the prisms; (iii) healing period, (iv) crack width during healing and (v) environmental conditions. The efficacy of the healing system was evaluated in terms of mechanical strength and stiffness recovery for samples subjected to three-point bend tests. Furthermore, a series of damage-loading cycles were performed on samples in order to explore the potential for multiple healing events.

The structure of the remainder of this paper is as follows:

Section 2 describes the development of the MVN units, including 3-D printing parameters, printing materials, form of the ligaments and MVN units, as well as the selection of an appropriate healing agent.

Section 3 presents the main experimental programme, with details of the final MVN design, experimental procedures and materials, and a description of the testing arrangements.

Section 4 presents the experimental results, discusses their significance and considers further avenues of research; some microscopy results conducted on fragments from crack surfaces; SEM/EDS analyses carried out on fragments collected from healed crack surfaces are also reported.

Section 5 draws some overall conclusions from the study.

## 2. MVN Design and Materials 

### 2.1. Design Concept and Considerations

**Figure 1:**
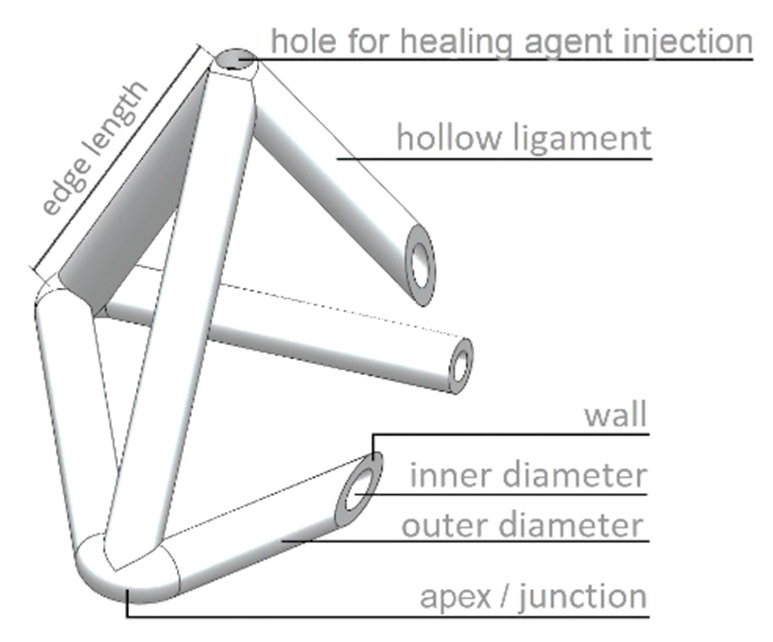
Schematic diagram of a generic MVN unit, main components.

The idea for the MVN presented in this paper (see Figure 1) came from the need to provide a practical and convenient method to supply sufficient healing agent to serviceability-sized cracks (e.g., 0.1 to 0.4 mm wide) in concrete structural elements that overcomes the problem of resistive capillary forces that bedevils closed tubular capsules. The proposed tetrahedral design addresses the latter issue because any crack plane crossing an MVN between the apices will fracture multiple ligaments thereby avoiding the possibility of negative pressures developing in the liquid healing agent. The MVN hollow ligaments are sized to ensure that there is sufficient healing agent to heal serviceability-sized macro-cracks. The advantages of the present tetrahedral MVN units are that, (i) they are (almost) directionally invariant with respect to their mechanical behaviour and, (ii) the apices anchor the unit within the concrete matrix, which limits the degree of sliding between the ligaments and the concrete matrix, and means that a ligament is likely to fracture when crossed by a concrete crack. The MVNs are sized to be approximately twice the size of the coarse aggregate particles. A tetrahedral shape is also advantageous because the ligaments are straight between the anchor points (apices), which means the ligaments are more likely to break when crossed by a crack. This is the main reason that the units are based on a tetrahedron rather than any other shape, e.g., sphere.

The additive manufacture of the MVNs allows the selection of the most suitable plastic filament to achieve the required MVN properties. The design of the ligaments and selection of the material used to form the MVNs are now considered in the following sub-sections. 

### 2.2. MVN Ligament Design

For reference, a schematic diagram of a generic MVN unit is given in Figure 1, which is used to identify the components of a typical MVN.

The successful design of MVNs is achieved when the following conditions are satisfied; (i) they survive the mixing process; (ii) they have sufficient bond and anchorage to minimise slip between the MVN ligaments and the concrete matrix, and to promote ligament rupture under the required conditions (see (iii)); (iii) the mechanical properties are such that ligaments rupture when crossed by a serviceability-size macro-crack; and (iv) they release sufficient healing agent to heal a serviceability-size crack in the vicinity of the MVN unit.

The primary aim of the first part of the research was to design the MVN ligaments such that they rupture at the desired crack opening value. In order to investigate the main parameters affecting the mechanical behaviour of the 3D printed MVNs (material, tube diameter, wall thickness, rib profile), a series of direct tension, pull-out and 3-point bend tests were conducted on solid bar and tubular specimens. The design criterion for first tube rupture is presented in the Appendix A of this paper, together with a full description of the experimental arrangements, and the presentation and discussion of the results. For completeness, the key outcomes from the study into the mechanical behaviour of the MVN’s can be summarised as follows:

- Both clear and white PLA (Polylactic acid) materials are suitable for the MVNs although clear PLA tubes are more likely to rupture at the required serviceability crack opening.
- Spiral rib patterns provide better bond than annular rib patterns and therefore are more likely to result in tube rupture at the required crack opening, with both 3 mm and 5 mm pitch rib patterns being viable, although 5 mm are preferred from a 3D-printing viewpoint.
- Rupture at the required crack opening is more likely to occur in thinner walled tubes (0.25 mm), although these have a greater permeability than thicker walled tubes (0.5 mm) (see discussion in Appendix A.1).

These conclusions informed the choice of MVN ligaments used in the main test series (See Table 1).

### 2.3. Encapsulation Environment and Selection of Healing Agents

In order to establish the best prophylactic agent to be stored into the MVNs, the encapsulation environment and MVN sealing techniques, a number of viability tests were performed on cylindrical tubes (inner diameter = 2.5 mm, length 30 mm). Three different CA agents: (a) Procure PC20, of low viscosity (5 cPs¹); (b) Procure PC60, of medium viscosity (80–120 cPs¹); (c) Procure PC90, of low to medium viscosity (34–44 cPs¹) were considered. The test details and results are summarised in Appendix A.2 of this paper.

PC60 was the only CA to provide evidence of an ability to remain liquid after 72 h; however, upon closer inspection it was apparent that the curing process of this CA had commenced and the viscosity of the PC60 at the end of the testing time was greater than that at the beginning. It is concluded that cyanoacrylate has the advantages of a one-component adhesive i.e., no mixing of components required and a long shelf life when encapsulated [29]. Unfortunately, due to its fast reaction upon contact with moisture or air, it undergoes premature curing inside the macro-capsules.

The presence of moisture in PLA, as a consequence of the hydroxyl group in the PLA molecular structure, initiates curing, and makes CA unsuitable for use as a healing agent in this particular experimental investigation.

As described in the previous section, sodium silicate solution (SS) is widely used as a healing agent in concrete and when in the presence of water it reacts with the calcium hydroxide present in cementitious materials to produce C-S-H gel which fills and heals cracks [30]. Additionally, it provides a better long-term compatibility with the cementitious matrix due to its ability to react chemically with the products of cement hydration. The slow reaction rate and stability of SS during encapsulation make it a promising healing agent candidate in MVN applications. A longevity test, similar to that reported in the Appendix A, was performed on 3D printed cylinder micro-capsules filled with SS, without any preventive surface coating treatments. The results confirmed that the SS was able to remain in a liquid state for 2 weeks before being cast in concrete. Moreover, no reactions between the SS and the PLA were noted, and there was evidence of liquid sodium silicate within the TETs upon breaking the concrete samples 3 months after casting.

## 3. Experimental Materials and Methods

### 3.1. MVN Design

The development of MVNs with a 3D tetrahedral shape—so called TETs—involved three variants. The TETs were printed from PLA using an Ultimaker2® printer (Utrecht, The Netherlands) with a 0.25 mm nozzle. The properties of the TETs are summarised in Table 1. The printing process of the TETs is complicated by the presence of ligaments placed on different geometrical planes, which leads to inherently anisotropic properties in the TETs, as well as difficulty in creating solid ligament walls by means of just overlapping PLA layers during printing.

**Figure 2:**
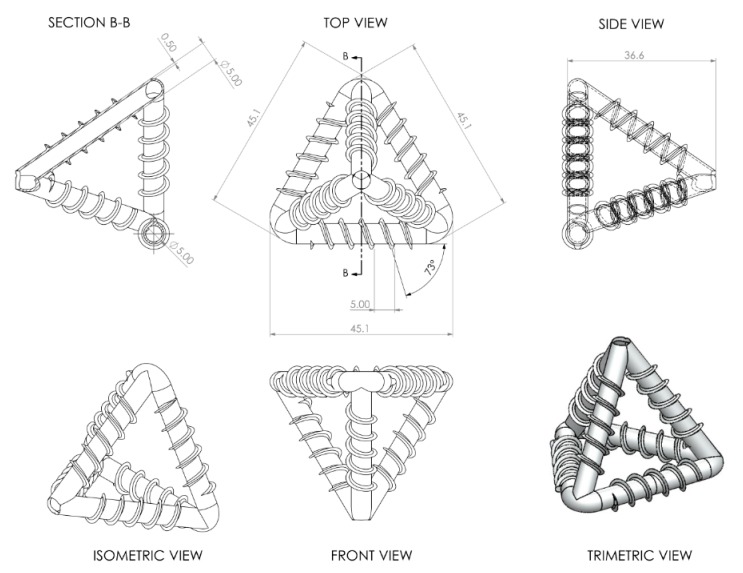
Dimensions of a TET unit (dimensions in mm).

Variant III provided the most promising results of the three variants, taking into account all of the required properties, i.e., an effective printing process, good bond strength and ligament wall impermeability. In order to mitigate the effect of different layer orientations on mechanical properties, a printing angle of 15° to the horizontal was found to provide an acceptable compromise between the printing duration, the number and form of internal supports and the effect of different building angles on the tensile strength and elastic properties of the ligaments of the MVNs. A low printing speed, together with the creation of layers via double printing helped to minimise the presence of micropores and achieve a completely impermeable unit. The dimensions of the Variant III TETs are provided in Figure 2.

### 3.2. Experimental Details

#### 3.2.1. Mix Details 

**Figure 3:**
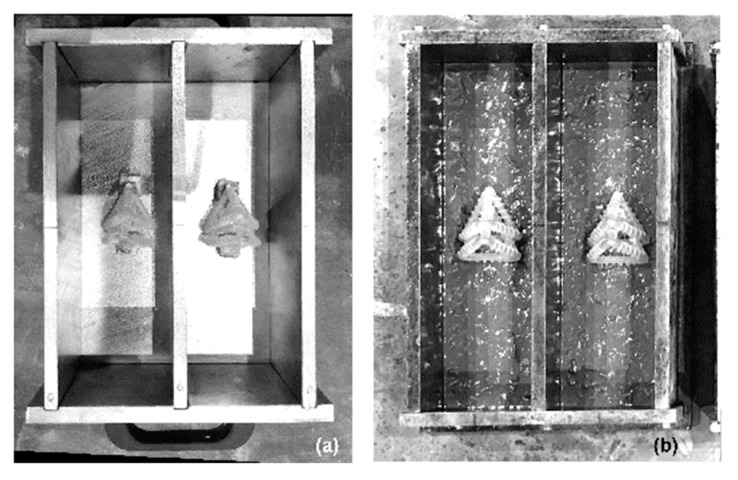
TETs disposition within the concrete beam (**a**) 2TETs placed via 10 mm spacer; (**b**) specimens filled to 10 mm from the base of the mould.

The mix constituents and proportions of the concrete comprised: Rapid Hardening Portland Cement (RHPC) (562 kg/m^3^); 0–2mm dried fine aggregate sand (562 kg/m^3^); 0–10 mm crushed limestone coarse aggregate (1124 kg/m^3^); and water (253 kg/m^3^). The same mix proportions, constituents and mixing protocol were used throughout the duration of the experimental programme. The dry materials (coarse aggregate, fine sand, cement) were mixed for 30 s, at which point approximately 3/4 of the water was added while the mixer was rotating. After a further 90s the remaining water was added, and mixing was stopped after a total of 210 s. The moulds were filled in 3 layers, with the prism specimens filled to 10mm from the base of the mould, allowing the TETs to be placed before being covered with additional concrete; TETs were manually placed in the centre third of a concrete prism mould (75 mm × 75 mm × 255 mm) directly in contact with the mould (Group 1) or fixed via 10 mm thickness concrete spacers secured to the bottom face of the mould (Group 2 and Group 3), as can be seen in Figure 3.

Samples were vibrated for 30 s each time to a maximum of 45 Hz prior to the TETs being placed, reducing to a maximum of 30 Hz once the TETs were placed inside the prism specimens to prevent excessive movement or damage to the TETs. For 6 h after casting the prisms were kept in the moulds covered with damp hessian; after this, samples were demoulded and placed in a water tank (20 °C ± 5 °C) for 24 h. A 5 mm central notch was created on the lower surface in order to induce a single vertical crack during the 3-point bending testing stage. Unless stated otherwise, all TETs were filled with sodium silicate (Na_2_O_3_Si) solution (d = 1.5 g/mL).

**Figure 4:**
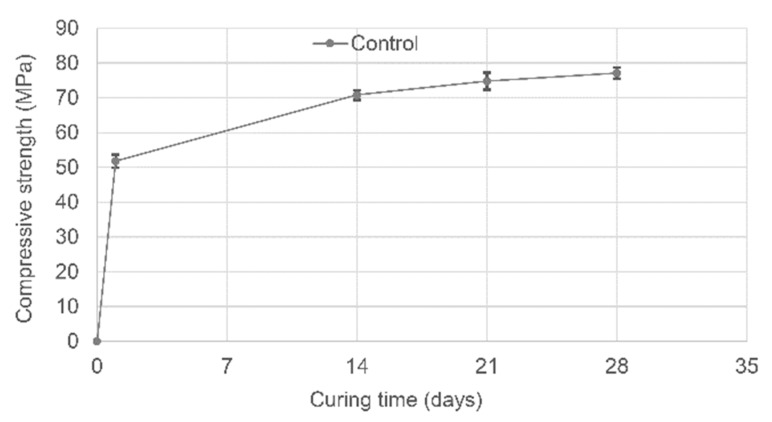
Concrete compressive strength variation with time.

Compression tests were conducted on three 100 mm cubes at 1, 14, 21 and 28 days in accordance with BS EN 12390-3:2009. The average compressive strength at 28 days was 77.1 MPa (CoV 2%). For the sake of completeness, the variation of the concrete compressive strength with time is represented in Figure 4. It is worth noting that at 1 day, when the pre-cracked phase takes place, the concrete has already reached 67% of its 28-day compressive strength.

#### 3.2.2. Experimental Arrangement 

**Figure 5:**
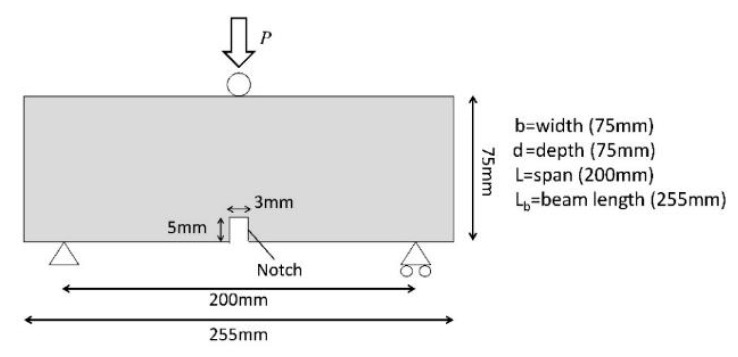
The three-point bend test arrangement.

After 24 h of curing, prisms were loaded until failure (control series) or until a Crack mouth opening displacement (CMOD) of 0.2 mm was recorded, using a 3-point bending test in accordance with BS EN 12390-5. A CMOD rate equal to 0.0001 mm/s, was used throughout the test. The experimental arrangement is illustrated in Figure 5. After the pre-cracking phase, samples were placed in laboratory environmental conditions (20 °C ± 5 °C, RH ~ 45%) for 14 days and then either reloaded until failure or reloaded until a CMOD of 0.2 mm was reached. The latter was used to examine the response of the specimens to multiple damage/healing cycles.

#### 3.2.3. Experimental Programme

The experimental programme, as presented in Table 2, can be separated into 3 main groups. The aim of Group 1 was twofold; to explore the influence of the TETs on the mechanical performance of the prismatic concrete beams and to evaluate the efficacy of the TETs in delivering the healing agent. The Group 2 test series was undertaken to explore cyclic damage-healing behaviour via 3 damage-healing cycles. Finally, the aim of the Group 3 test series was to explore a range of variables that may affect healing efficacy, such as:

- the level of damage: the effect of the damage was studied by loading the samples until a crack opening of 0.4 mm was achieved;
- the amount of water available for the reaction between SS and CH to form a C-S-H gel: this was studied by placing the specimens in a sealed container covered with a damp hessian cloth to provide high relative humidity values (>90%) at a constant room temperature (ca. 20 °C) following the pre-cracking phase;
- the healing period: the time of healing after the pre-cracking phase was increased to 21 days.

The sample designation conforms to the following: V_W_X_Y_Z; where V is the Group number; W is the number of embedded MVNs (C indicates plain concrete); X is the age of the first test; Y is the healing agent used to fill the MVNs (SS = sodium silicate) or empty MVNs; Z is additional information regarding the specimen (0.4 = CMOD (mm), H = high moisture curing condition, 21= healing period of 3 weeks).

## 4. Experimental Results

The results of the pre-cracked and post healed specimens are presented in terms of load vs CMOD responses. Comparison of the pre-cracking and post-healing responses with those of the control specimens, allows the degree of healing to be calculated. The healing indices, assessing the influence of healing on the recovery of the load-bearing capacity as well as on the flexural stiffness and fracture energy are discussed herein.

The nominal flexural stress is obtained from the following Equation (1).

(1) $$\sigma^{k}=\frac{3\mathrm{Pl}}{2{\mathrm{bd}}^{2}}$$

where P is the peak load, l is the span (200 mm), b is the width of the concrete samples (75 mm) and d is the depth (75 mm).

The authors recognise that Equation (1) does not give the true value of the stress once micro-cracking has started; nevertheless, it does provide a useful measure for evaluating and comparing the degree of healing in different specimens. For completeness, the full experimental results are also presented as Load/Displacement responses.

**Figure 6:**
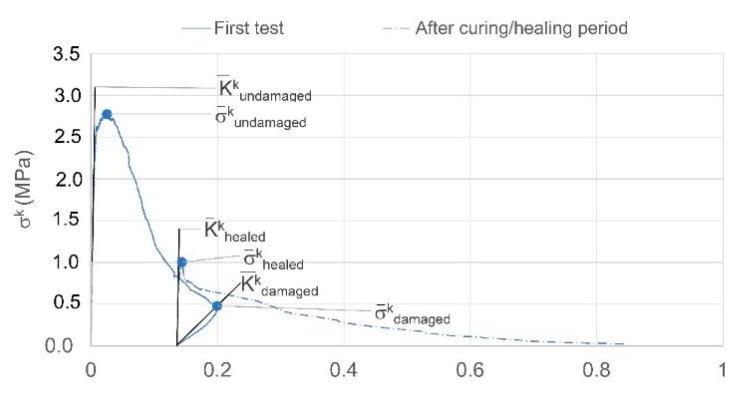
Stress-CMOD curves before and after healing: notation and definition of the parameters for the indices of healing.

As represented in Figure 6, the effectiveness of healing can be evaluated by calculating the strength gained after the healing period $\left({\bar{\sigma }}_{\mathrm{healed}}^{k}\right)$ with respect to the residual strength measured at the maximum pre-cracked opening $\left({\bar{\sigma }}_{\mathrm{damaged}}^{k}\right)$ and comparing it to the maximum stress exhibited by the same specimens in undamaged conditions $\left({\bar{\sigma }}_{\mathrm{undamaged}}^{k}\right)$. With reference to the notation in Figure 6, the index of strength recovery $\eta_{\sigma }^{k}$ (%) is defined as in Equation (2) [4,31].

(2) $$\eta_{\sigma }^{k}=\frac{{\bar{\sigma }}_{\mathrm{healed}}^{k}-{\bar{\sigma }}_{\mathrm{damaged}}^{k}}{{\bar{\sigma }}_{\mathrm{undamaged}}^{k}-{\bar{\sigma }}_{\mathrm{damaged}}^{k}}$$

Moreover, the loading/unloading/reloading cycle allows the evaluation of the recovery of flexural stiffness as a result of healing. This is calculated using the undamaged stiffness—determined from the slope of the linear part of the load-CMOD curve in the first cycle $\left({\bar{K}}_{\mathrm{undamaged}}^{k}\right)$, the value of the secant unloading stiffness $\left({\bar{K}}_{\mathrm{damaged}}^{k}\right)$, and the tangent reloading stiffness in the post-healing curve $\left({\bar{K}}_{\mathrm{healed}}^{k}\right)$, a linear curve fitting function was used to compute the stiffness and the index of flexural stiffness recovery $\eta_{K}^{k}$ (%) was calculated as in Equation (3) [32].

(3) $$\eta_{K}^{k}=\frac{{\bar{K}}_{\mathrm{healed}}^{k}-{\bar{K}}_{\mathrm{damaged}}^{k}}{{\bar{K}}_{\mathrm{undamaged}}^{k}-{\bar{K}}_{\mathrm{damaged}}^{k}}$$

**Figure 7:**
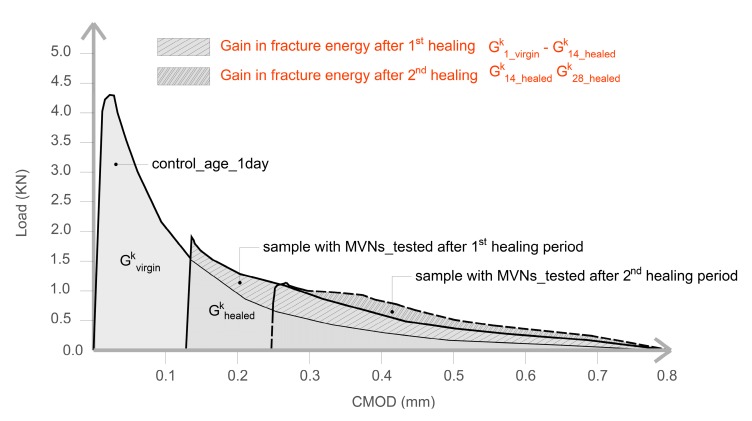
Schematic Load-CMOD diagram, notation and definition of the parameters for the index of fracture energy recovery.

For the sake of completeness, the fracture energy for samples tested in flexure has been also evaluated. The recovery of fracture energy is calculated as the difference between the area subtended by the Load-CMOD curve of control samples ${\bar{G}}_{1\_\mathrm{virgin}}^{k}$ and the area subtended by samples with TETs tested after the healing period $\left({\bar{G}}_{\mathrm{healed}}^{k}\right).$ The fracture energy recovery index, $\eta_{\mathrm{Gf}}^{k}$ (%) is calculated as in Equation (4) and as illustrated in Figure 7.

(4) $$\eta_{\mathrm{Gf}}^{k}=\frac{{\bar{G}}_{1\_\mathrm{virgin}}^{k}-{\bar{G}}_{\mathrm{healed}}^{k}}{{\bar{G}}_{1\_\mathrm{virgin}}^{k}}\times 100\%$$

### 4.1. Group 1 Results

**Figure 8:**
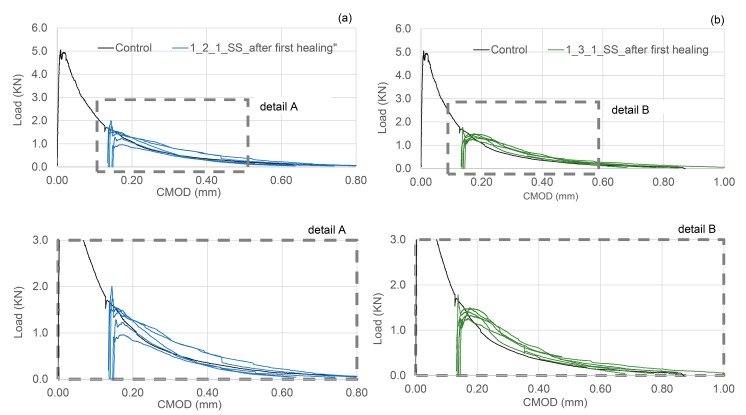
First Damage/Healing flexural load-CMOD responses for control specimens and specimens containing SS filled TETs (**a**) 2 TETs and (**b**) 3 TETs.

In Table 3 the results of the flexural tests are shown for the different tested samples, together with the indices of healing derived from the load-CMOD responses. The results shown in Table 3 are the average of nominally identical specimens. The full load-CMOD responses for the tests for SS-filled TETs are given Figure 8.

**Figure 9:**
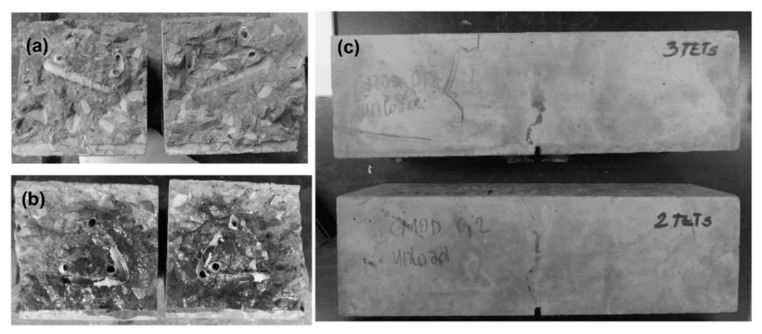
Group 1: (**a**) samples with empty TETs after breakage; (**b**) samples with 2 TETs filled with SS after breakage; (**c**) evidence of TET breakage and SS release after the pre-cracking phase.

It can be seen that the presence of TETs marginally reduces the flexural strength of the specimens tested after 24 h of curing. The specimens with 2 TETs filled with SS show an average initial flexural strength approximately 9% less than the control samples. The initial flexural strength seems to decrease further (~13%) when the number of TETs is increased to 3, and furthermore when the TETs are empty (~16%). It can be reasonably assumed that TETs act as inclusions in the concrete, which introduces inhomogeneities into the matrix. Moreover, the gentle vibration adopted may not be sufficient to achieve proper compaction of the mixes and will result in a reduction of the bond stress between the concrete and the TETs [33]. The reduction in flexural strength may be influenced by the positioning of the TETs within the prism cross section. The tendency of the TET’s to float upwards in the section was also noted and was particularly prevalent in the prisms containing empty TETs (see Figure 9a). At the end of the test, the average distance between the first broken ligaments of TETs and the bottom of the prisms was recorded as 14.4 mm and 18.6 mm in samples with 2 and 3 TETs respectively (Figure 9b). Since a tapered crack is assumed then the CMOD at the level of TETs will also vary, which may influence the propensity of the TET ligaments to rupture and ultimately affect the efficiency of the healing system. The use of spacers in Group 2 addressed the variation in TET positioning. As expected for the control samples, a low strength and stiffness recovery, of 2% and 8% respectively, was observed at the end of the re-loading phase. Given the short healing timescales employed in the current study (14 days), it may well be expected that the level of autogenous healing observed in specimens is limited. At a crack width of 0.2 mm, visual observations clearly showed an indication of the breakage of the TETs and the discolouration of the concrete surface confirmed the release of the SS (see Figure 9c). The system of interconnected channels of the TETs provides an effective reservoir of healing agent that facilitates the flow of SS into cracks under capillary action. Specimens containing TETs filled with SS, pre-cracked and exposed to laboratory conditions for 14 days, gave positive healing results, with a load recovery index of 16% for the samples with 2 TETs and 12% for samples with 3 TETs, whilst the stiffness recovery indices were found to be 73% and 67% respectively. At first sight, increasing number of TETs does not appear to have a positive effect on the flexural strength after healing; this suggests that not all the TETs were intersected by the crack and thus not all of the SS stored inside the TETs contributed to the healing process. Nevertheless, when considering the fracture energy (Figure 7) the recovery index underlines the positive influence of a greater number of TETs within the concrete, increasing from 36% to 85% when 3 TETs are employed as opposed to just 2.

### 4.2. Group 2 Results

**Figure 10:**
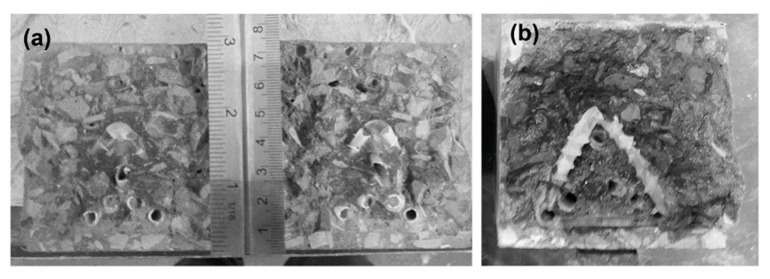
Group 2: (**a**) spacers blocks employed to control the movement of the TETs; (**b**) expansion area of the healing agent.

Spacers were used to control the cover to the TETs, such that the influence of the position of the TETs in the cross section could be eliminated, as confirmed in Figure 10. Moreover, in all the series of Group 2 three damage-healing cycles were performed on control samples as well as on samples containing TETs, in order to better understand the ability of the TETs to store sufficient healing agent for multiple healing events. The results of flexural tests performed on prismatic concrete specimens in Group 2 are detailed in Table 4 and the associated healing indices for each cycle are reported in Table 5.

**Figure 11:**
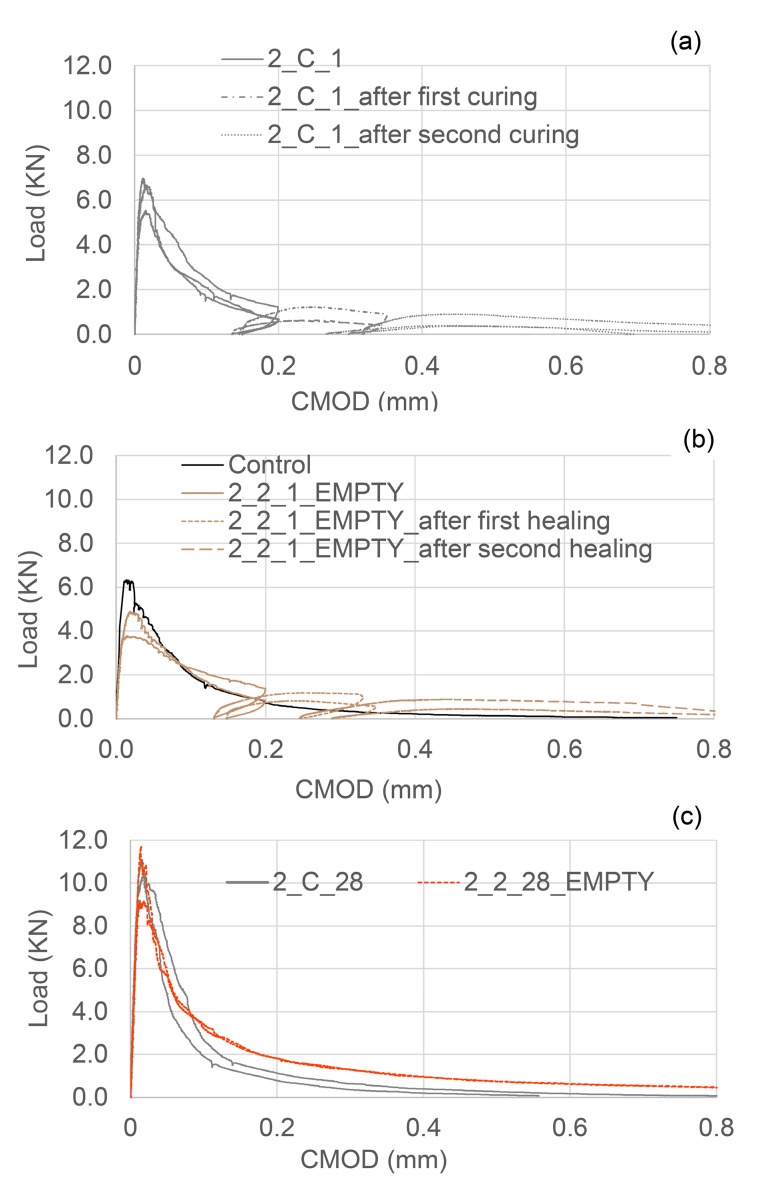
(**a**) Flexural load-CMOD response of control specimens; (**b**) Flexural load-CMOD response for 2 damage-healing cycles of specimens containing 2 empty TETs; (**c**) Flexural load-CMOD response at 28 days of specimens with and without empty TETs.

For the sake of completeness, the behaviour of the compressive strength of the control cube specimens is reported in Figure 4. As expected for RHPC specimens, 70% of the compressive strength was reached after 24 h, while after 14 days of curing (even in laboratory ambient conditions) 90% of the 28-day strength had been achieved. Control specimens tested in flexure exhibited the same behaviour as observed in the Group 1 control specimens, with minimal healing observed in the first and second healing cycles (Figure 11a,b). The limited availability of unreacted cement particles to facilitate healing can be attributed to the high early strength gain due to the use of RHPC. The inclusion of spacers in the control beams results in a flexural strength loss of approximately 8.5%. 

Contrary to the trend observed in the difference in flexural strength of beams with and without TETs at an age of 1 day, specimens cured until 28 days (1 day water curing followed by 27 days in ambient laboratory conditions) obtained very similar strengths. Moreover, as can be seen in the flexural load-CMOD responses represented in Figure 11c, the presence of TETs seems to bridge cracks when the majority of the concrete ligaments in the matrix are lost. It is reasonable to assume that—thanks to the prolonged curing period—the adhesion between the TETs and the concrete matrix has been improved, allowing the TETs—even if empty—to act as reinforcement. This also suggests that the presence of empty TETS, after healing agent release, is not detrimental to the strength of the concrete matrix.

**Figure 12:**
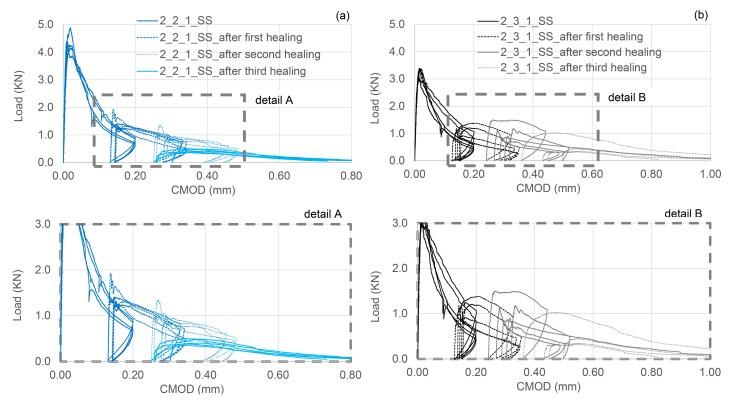
Flexural load-CMOD responses for 3 damage-healing cycles for specimens containing (**a**) 2 TETs filled with SS; (**b**) 3 TETs filled with SS.

The complete damage-healing cycles of concrete specimens containing 2 TETs and 3 TETs filled with SS are represented in Figure 12a,b respectively. After the first healing period, the load recovery index was 19% for sample with 2 TETs and 20% for sample with 3 TETs, while the stiffness recovery indices were found to be equal to 75% and 73% respectively. A positive healing result has been maintained after the second healing period when the load recovery index is 11% in both cases and the stiffness recovery indices were 40% and 33% for specimens with 2 and 3 TETs respectively. The index of fracture energy recovery has confirmed the efficiency of the TETs system, resulting in 47–48% recovery even after the second healing cycle.

After the third damage-healing cycle, the concrete-TETs-system appears to have consumed the reservoir of healing agent in the TETs. The strength recovery index is negligible, nonetheless a recovery of stiffness is still notable: 5% and 9% in specimens with 2 and 3 TETs respectively. It is worth highlighting that a small amount of liquid SS was found within the units after breaking the specimens containing 3 TETs, while the SS was almost all solid/dried in specimens containing 2 TETs. Moreover, the crack plane coverage of SS in the cross-section approached 100% in all the specimens tested, as can be seen in Figure 10b.

When the TETs system is well placed inside the concrete beams and fixed into position, all the healing indices have pointed towards a performance better than the control specimens in Group 2 and all specimens in Group 1. Moreover, the number of SS filled TETs contained within the specimens does not seem to significantly alter their healing behaviour. The TET units have been shown to be a perfusable vascular system [34] that acts as a discrete reservoir of healing agent that is available for multiple healing events. Furthermore, the system has been proven to react to rapid damage (in short-term) and heal multiple occurrences of damage that arise from cyclic loading (medium-long term damage sequences), albeit with the appropriate selection of healing agent.

### 4.3. Group 3 Results

**Figure 13:**
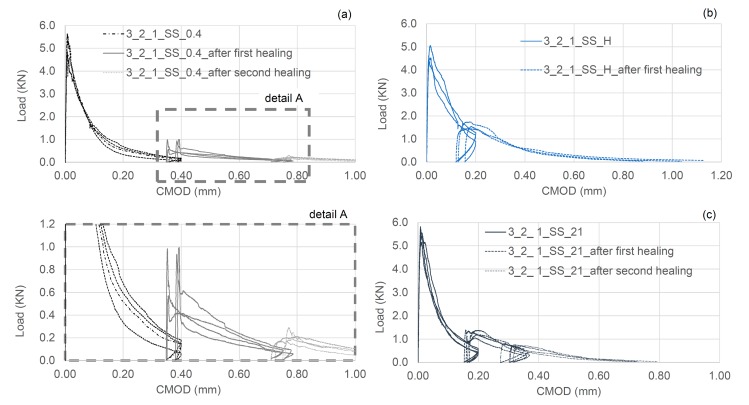
Group 3: (**a**) Flexural load-CMOD response for specimens containing 2 TETs, CMOD = 0.4 mm; (**b**) Flexural load-CMOD response for specimens containing 2 TETs filled with SS cured in high moisture conditions; (**c**) Flexural load-CMOD response for specimens containing 2 TETs filled with SS and stored for an extended healing period.

In the first series of tests in Group 3 the effect on the healing response of varying the degree of damage was evaluated using a flexural test in which the final CMOD was 0.4 mm. As can be seen in the load-CMOD curves given in Figure 13a, the residual flexural strength at the end of the pre-cracking phase is negligible (~100 N). Interestingly, concrete samples containing 2 TETs filled with SS, showed a recovery of strength and stiffness of 17% and 35% respectively. Both the healing indices are positive and very close to cases presented in the previous section where the CMOD after the pre cracking stage was only 0.2 mm. The results of the flexural tests performed on samples of Group 3 are summarised in Table 6.

High moisture conditions during the healing phase appear to moderately decrease the healing efficacy in terms of strength and stiffness; however, the recovery of fracture energy is still positive (Figure 13b). The findings from these samples suggest that in laboratory ambient conditions, the matrix has a sufficient supply of water available for the reaction between SS and CH to form a C-S-H gel. The continuous provision of additional water, throughout the healing period, may have diluted the SS, thus affecting its efficacy as a healing agent. The aforementioned reaction between SS and CH in the concrete matrix-TETs system takes approximately 14 days, and a prolonged period of healing is of limited benefit (Figure 13c).

When comparing all of the specimens tested with SS in this experimental programme there appears to be a ceiling value of 20% recovery of flexural strength after the first loading cycle. This value then diminishes with further loading cycles, which may be attributed to depletion of the sodium silicate in the TETs and repeated damage of the “healed” material, which would appear to be weaker than the host cementitious matrix. Flexural stiffness recoveries similarly decrease with consecutive loading cycles although still remains above 5–10% after 3 loading cycles. 

### 4.4. SEM/EDS Analyses

In order to provide a deeper insight into the mechanical behaviour of concrete containing SS filled TETs, the chemical composition of the healing products, as generated by different damage-healing events, were characterized by means of SEM/ EDS analysis.

Light microscopy observations were carried out on the crack surfaces of samples containing 2 TETs (Group 2) at the end of the first damage-healing cycle using a Leica MZ6 stereo microscope equipped with a digital camera. Fragments collected from crack surfaces were also analysed with scanning electron microscopy (SEM). The latter was carried out with a Zeiss Sigma|VP (Jena, Germany), a variable pressure instrument (VP-SEM) equipped with a Bruker Quantax 200 energy dispersive x-ray spectroscopy (EDS) system (Bruker, Billerica, MA, USA).

**Figure 14:**
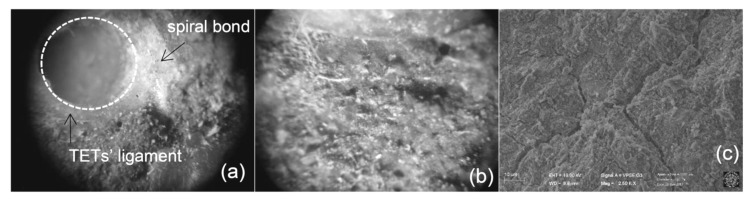
Micrographies of the crack surface; (**a**) TETs’ ligament detail observed with light microscopy; (**b**) presence of white spread crystal close to TETs’ ligaments observed again with light microscopy; (**c**) SEM image of fragments collected from crack surface.

The 3D printing process has enabled the production of TET units with fully bonded layers; this yields good inter-layer adhesion, as can be seen in Figure 14a, which assists in achieving TET strength and impermeability. Moreover, few voids are visible at the interface between TETs’ ligaments and concrete matrix, indicating a good physical bond to the cement matrix. As noted previously, the main mechanism of self-healing, with SS as a healing agent, is the reaction between the SS in dissolved form and CH, through the exchange of calcium cations. This is also accompanied by crystallisation of surplus SS when the water of the solution evaporates [20]. White crystals, compatible with this reaction, are present in the region closest to the TET ligaments (Figure 14b) and SEM observations has highlighted their homogeneous release into the concrete matrix (Figure 14c).

**Figure 15:**
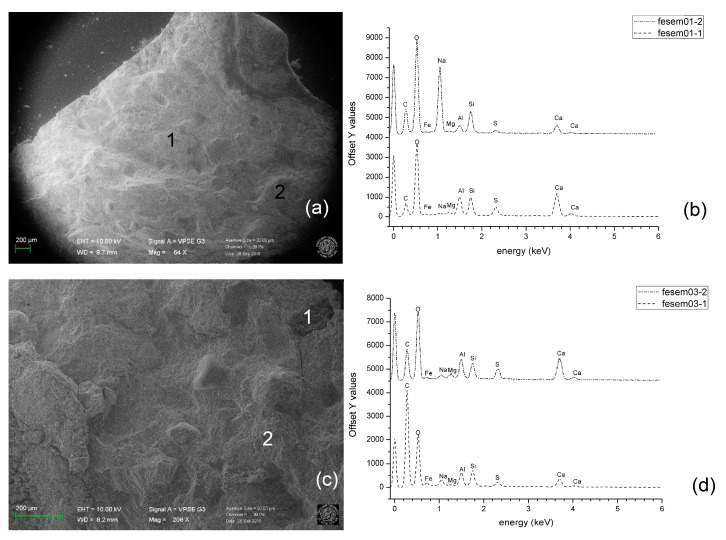
(**a**) SEM image of fragment collected along the crack surface after the first healing cycle; (**b**) EDS at point 1 and 2; (**c**) SEM image of fragment collected along the crack surface after the third healing cycle; (**d**) EDS at point 1 and 2.

A deeper insight into the results is provided by SEM/EDS analysis. SEM observation (Figure 15a,b) showed the main morphology of the matrix after the first damage-healing cycle. The EDS microanalyses were performed on two different points, chosen at random on the crack surface. 

The main chemical elements observed on the crack surface are Si, O, Ca. However, the comparison between the EDS spectra (Figure 15b,d) highlights the varying spatial distribution of these elements on the crack surface in the same healing cycle and between healing cycles. The presence of moderate Ca peaks and limited Na peaks in the majority of measurements, points towards the consumption of SS and formation of C-S-H gel in the damaged area [35]. For point 2 in the first healing cycle, the large Na peak indicates crystallisation of the SS on the crack face. This may be attributed to the abundance of SS released during the first cycle and a finite quantity of CH available for the reaction on the crack surface. The absence of Na peaks on the third healing cycle (Figure 15d) suggests new crack formation and exposure of additional CH to the remaining SS released from the TETs. Mechanical tests have confirmed that flexural strength and stiffness recovery in the third cycle is small and the absence of excess SS on the crack face supports these findings.

### 4.5. Summary Remarks 

In this experimental programme, the effectiveness of the self-healing approach is limited by the properties of the healing agent, particularly because an ideal healing agent should remain active, even if in a dormant state, for several years. The success of the healing system is strongly related to the strength of the healing agent and to the bond strength between the healed material and the concrete matrix. If successful, the strength of the repaired crack should be similar to that of the surrounding concrete matrix, if not, further cracks may occur in the same location again, where the healing agent may have already been consumed. The release of a healing agent with a high stiffness when cured also allows the concrete to regain stiffness, thanks to a good stress transfer capability; however, this may also lead to a less flexible system which is not able to accommodate further movement or damage. Single-component agents are usually preferred to dual-component agents, due to reports of incomplete mixing of the different components; nevertheless, attempts to use air cured cyanoacrylate have resulted in premature curing of the agent during encapsulation. SS solution has a viscosity suitable for being released by the TETs.

## 5. Conclusions

In this research the behaviour of an innovative self-healing system has been investigated for applications in concrete. It involved the use of newly designed 3D printed MVNs, in the shape of regular tetrahedrons with hollow ligaments. Preliminary tests were carried out in order to achieve the optimum form of the MVNs to include: the optimum ligament dimensions (to maximise the healing agent volume); the optimum surface profile (to achieve enhanced bond stress with the matrix); their optimum distribution within the mix; and the best mechanical behaviour which guarantees survival during the mixing phase and brittleness for rupture during damage. Studies on the best prophylactic agents to use have also been conducted. The results before and after the healing stages were evaluated by means of a three-point bend test, and the performance of the prismatic concrete samples containing TETs filled with SS were compared to the reference control samples. The proposed methodology of analysis, which encompasses a wide range of parameters, i.e. the number of TETs and their position within the concrete beams, exposure conditions and durations and level of the damage provides a reference for future research. From the analyses of the results presented in the paper, the following main conclusions behave been drawn:

- The 3D printed Mini Vascular Networks (MVNs) form the basis of a viable self-healing system for concrete.
- Clear PLA is the most appropriate material for the MVNs, from amongst the 3D printed plastic filament materials tested.
- A spiral bond pattern with 5 mm pitch ribs provides sufficient bond between the MVN ligaments and the concrete matrix to ensure that the hollow ligament tubes rupture when crossed by a serviceability-size concrete crack.
- A double thickness (2 mm × 0.25 mm) ligament wall provides the best balance between impermeability and strength; the former being required to prevent premature healing-agent curing and the latter being such as to ensure rupture at the appropriate crack opening.
- The MVNs store sufficient healing agent to cure serviceability-sized cracks in concrete.
- The MVN system of interconnected channels overcomes the problems associated with capillary suction forces that prevent healing agents being released from closed-end tubular capsules.
- MVNs filled with SS can produce strength and stiffness recoveries of 20% and 75–80% respectively in prismatic concrete beams.
- The presence of MVNs has little effect on the pre-healed strength of concrete elements in which they are embedded.
- MVN systems filled with SS can heal multiple occurrences of damage, with (representative) strength and stiffness healing recoveries of 20%, 11%, 0% and 80%, 40% and 5% respectively for the first, second and third loading cycles.
- Samples with a high level of damage (i.e., a CMOD of 0.4 mm), characterised by a negligible residual flexural strength after the pre-cracking phase, exhibited significant healing behaviour; with strength and stiffness recoveries of 17% and 35% respectively.

On-going research is exploring TET designs for multi-component adhesives, which guarantee air and water tightness. In this context, healing agents with expansive curing actions will be considered for the recovery of mechanical properties of the host specimen. The development of enhanced prophylactic agents will also provide the potential for agents with greater encapsulation stability and a range of trigger mechanisms for curing.

## Figures and Tables

**Table 1:** TET properties.

Variant	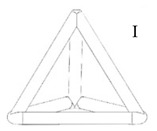	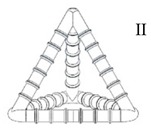	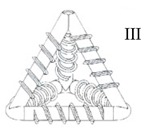
PLA filament	white	white/clear	clear
Printing orientation (ϑ)	flat	flat	Axis x + 15°; y + 15°
Outer wall print speed	60 mm/s	50 mm/s	40 mm/s
Printing temperature	210 °C	200 °C	195 °C
Ribs	-	Annular	Spiral
Edge length (mm)	40	42	44
Wall thickness (mm)	1	0.5	0.5 × (0.25 × 2 ^a^)
Inner diameter (mm)	3	3.5	5
Ink evidence during the flexural test	no	no	yes
Volume of healing agent	1.7 cm^3^	2.4 cm^3^	5.2 cm^3^
Impermeability	no	no	yes

^a^ denotes a double-printed wall to give a total wall thickness of 0.5 mm.

**Table 2:** Details of the experimental programme.

	Sample Designation	No. Samples	No. TETs	Healing Agent	CMOD (mm)	First Curing/Healing Period (days)	CMOD (mm)	Second Curing/Healing Period (days)
GROUP 1	1_C_1	3	0	-	-	1	-	-
	1_C_1	3	0	-	-	14	-	-
	1_2_1 EMPTY	3	2	-	-	14	-	-
	1_2_1_SS	5	2	SS	0.2	14	-	-
	1_3_1_SS	5	3	SS	0.2	14	-	-
GROUP 2	2_C_1	3	0	-	0.2	14	0.2	14
	2_C_28	3	0	-	-	28	-	-
	2_C_1 ^s^	3	0	-	-	14	-	-
	2_2_28 EMPTY ^s^	3	0	-	-	28	-	-
	2_2_1_EMPTY ^s^	3	0	-	0.2	14	0.2	14
	2_2_1_SS ^s^	5	2	SS	0.2	14	0.2	14
	2_3_1_SS ^s^	5	3	SS	0.2	14	0.2	14
GROUP 3	3_2_1_SS_0.4	3	2	SS	0.4	14	0.4	14
	3_2_1_SS_H *	3	2	SS	0.2	21	-	-
	3_2_ 1_SS_21	3	2	SS	0.2	21	-	-

Note: ^s^ denotes the presence of spacers. * denotes high humidity placement following initial damage.

**Table 3:** Group 1: Flexural test results and indices of healing for one damage-healing cycle.

Group 1			First Healing Cycle			
	σ^k_1^_undamaged_ MPa (CoV%)	σ^k_1^_damaged_ MPa (CoV%)	σ^k_14^_healed_ MPa (CoV%)	$h_{s\_14}^{k}$	$h_{E\_14}^{k}$	$h_{Gf\_14}^{k}$
1_C_1	3.83 (5)	-	-	-	-	-
1_C_1	4.29 (2)	0.54 (12)	0.60 (22)	2	8	23
1_2_1_EMPTY	3.41 (3)	-	-			
1_2_1_SS	3.71 (12)	0.58 (22)	1.10 (24)	16	73	36
1_3_1_SS	3.52 (9)	0.70(7)	1.00 (12)	12	67	85

**Table 4:** Group 2: Flexural test results for three damage-healing cycles.

	Flexural Strength (MPa) (CoV%)							
			1^st^ Healing Cycle		2^nd^ Healing Cycle		3^rd^ Healing Cycle	
Group 2	σ^k_1^_undamaged_	σ^k_28^_undamaged_	σ^k_1^_damaged_	σ^k_14^_healed_	σ^k_14^_damaged_	σ^k_28^_healed_	σ^k_28^_damaged_	σ^k_42^_healed_
2_C_1	4.22 (10)		0.51 (12)	0.58 (22)	0.37 (39)	0.39 (45)	-	-
2_C_28	-	7.26 (8)	-	-	-	-	-	-
2_C_1 ^s^	3.86 (3)	-	-	-	-	-	-	-
2_2_28 EMPTY ^s^	-	7.40 (12)	-	-	-	-	-	-
2_2_1_EMPTY ^s^	3.41 (9)	-	0.9 (14)	0.9 (12)	0.5 (24)	0.47 (32)	-	-
2_2_1_SS ^s^	3.16 (7)	-	0.55 (17)	1.05 (16)	0.42 (18)	0.72 (17)	0.30 (27)	0.30 (12)
2_3_1_SS ^s^	3.22 (5)	-	0.67 (31)	1.19 (10)	0.48 (49)	0.78 (22)	0.39 (54)	0.38 (52)

Note: ^s^ denotes the presence of spacers.

**Table 5:** Group 2: Healing indices for three damage-healing cycles.

	1^st^ Healing Cycle			2^nd^ Healing Cycle			3^rd^ Healing Cycle		
Group_2	$h_{s_{14}}^{k}$ (%)	$h_{E_{14}}^{k}$ (%)	$h_{Gf_{14}}^{k}$ (%)	$h_{s_{28}}^{k}$ (%)	$h_{E_{28}}^{k}$ (%)	$h_{Gf_{28}}^{k}$ (%)	$h_{s_{42}}^{k}$ (%)	$h_{E_{42}}^{k}$ (%)	$h_{Gf_{42}}^{k}$ (%)
2_C_1	2	7	26	1	2	12	-	-	-
2_C_28		-	-		-	-	-	-	-
2_C_1^s^	-	-	-	-	-	-	-	-	-
2_2_28 EMPTY^s^		-	-		-	-	-	-	-
2_2_1_EMPTY^s^	0	0	-	0	0	0	-	-	-
2_2_1_SS^s^	19	75	-	11	40	47	0	5	24
2_3_1_SS^s^	20	79	-	11	33	48	0	9	44

Note: ^s^ denotes the presence of spacers.

**Table 6:** Group 3: Flexural test results, indices of healing for two damage-healing cycles.

		1^st^ Healing Cycle					2^nd^ Healing Cycle				
Group_3	σ^k_1^_undamaged_ (CoV%) (MPa)	σ^k_1^_damaged_ (CoV%) (MPa)	σ^k_14^_healed_ (CoV%) (MPa)	$h_{s_{14}}^{k}$ (%)	$h_{E_{14}}^{k}$ (%)	$\sigma h_{Gf_{14}}^{k}Z$ (%)	σ^k_14^_damaged_ (CoV%) (MPa)	σ^k_28^_healed_ (CoV%) (MPa)	$h_{s_{28}}^{k}$ (%)	$h_{E_{28}}^{k}$ (%)	$h_{Gf_{28}}^{k}$ (%)
3_C_1	4.02 (6)	-	-	-	-	-	-				
3_2_1_SS_0.4	3.73 (7)	0.08 (42)	0.69 (3)	17	35	-	0.05 (19)	0.16 (20)	3	4	8
3_2_1_SS_H *	3.31 (6)	0.76 (15)	1.12 (7)	14	40	89	-	-	-	-	-
	**σ^k_1^_undamaged_** **(CoV%)** **(MPa)**	**σ^k_1^_damaged_** **(CoV%)** **(MPa)**	**σ^k_21^_healed_** **(CoV%)** **(MPa)**	$h_{s_{21}}^{k}$ **(%)**	$h_{E_{21}}^{k}$ **(%)**	$h_{Gf_{2114}}^{k}$ **(%)**	**σ^k_21^_damaged_** **(CoV%)** **(MPa)**	**σ^k_42^_healed_** **(CoV%)** **(MPa)**	$h_{s_{42}}^{k}$ **(%)**	$h_{E_{42}}^{k}$ **(%)**	$h_{Gf_{42}}^{k}$ **(%)**
3_2_1_SS_21	3.96 (4)	0.33 (11)	0.90(9)	16	67	-	0.29 (29)	0.52 (8)	6	24	21

* denotes high humidity placement following initial damage.

**Figure A1:**
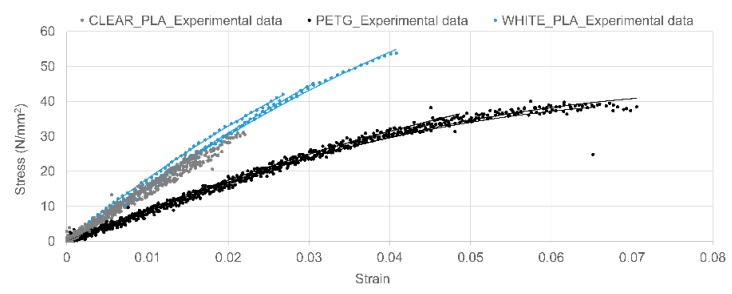
Stress-Strain graph of solid tubular specimens printed using clear PLA, white PLA and PE.

**Figure A2:**
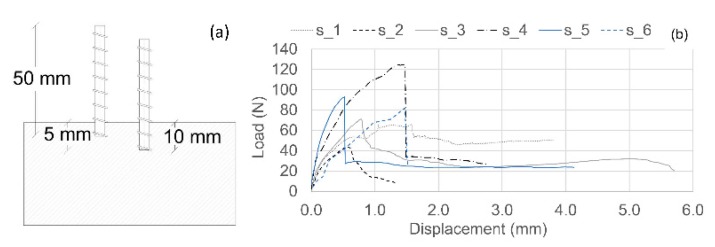
Pull-out test, (**a**) scheme of tubes inserted into mortar specimen; (**b**) representative Load-Displacement diagram.

**Figure A3:**
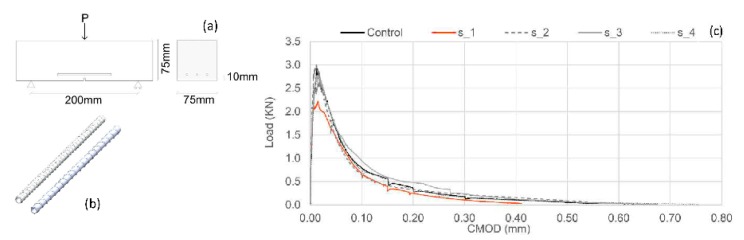
(**a**) Three-point bend test setup on samples containing 3 tubes. (**b**) Examples of tubes with spiral ribs (pitch 3 and 5 mm). (**c**) Load -CMOD diagram of samples containing hollow tubes.

## Appendix A

### Appendix A.1. MVN Ligament Design

The primary aim of the first part of the research was to design the MVN ligaments such that they rupture at the desired crack opening value. In order to investigate the main parameters affecting the mechanical behaviour of the 3D printed MVNs, tests were first conducted on solid bar specimens formed from the different materials and then on tubular specimens. The tests comprised the following;

1. Direct tension tests on 100 mm long 4 mm diameter bar specimens formed from different materials;
  - The materials investigated included two forms of polylactic acid, (PLA) (i.e., clear-PLA and white-PLA) and polyethene terephthalate glycol-modified, (PETG).
2. Three sets of tests using 3D-printed tubular specimens, namely; (i) direct tension tests; (ii) direct pull-out tests, in which a tensile load was applied to the free end of a tube partially embedded in a cementitious block, and (iii) three-point bend tests on cementitious beams with embedded tubes. The parameters varied in these tests were the following:
  - Filament material (PLA and PETG);
  - External diameter (4 or 5 mm);
  - Wall thickness (0.25 or 0.4 mm);
  - Rib profile and spacing (annular or spiral ribs, spacing 3 mm or 5 mm).

#### Appendix A.1.1. Quantification of the Desired Ligament Behaviour

The selected design criterion for first tube rupture is a crack opening (w_k_) of 0.15 mm in the cementitious matrix. It is assumed that the ribs anchor the tube either side of a crack such that the strain in the tube across a crack at rupture is approximately ε_tube_ = w_k_/l_ef_, where l_ef_ is taken as the rib spacing or pitch, and that rupture occurs when the following condition is satisfied;

(A1) $$\varepsilon_{\mathrm{tube}}>\varepsilon_{\mathrm{rup}}$$

where $\varepsilon_{\mathrm{rup}}$ is the rupture strain of the ligament material.

#### Appendix A.1.2. Direct Tension Tests on Solid Bar Specimens

The direct tension tests were undertaken on 100 mm length solid bar samples, using a servo-hydraulic universal machine with a 100 N load cell (max rating) at a displacement rate of 0.01 mm/min. Digital Image Correlation (DIC) camera (LaVision Imager X-lite 8M CCD camera, LaVision, Göttingen, Germany) was employed to measure the strain using a reference length of 25 mm. The results were processed and analysed by using DaVis software, 2015. The results from the tests are presented in Figure A1 and Table A1.

**Table A1:** 3D printed material properties: elongation at break, tensile modulus evaluation (Coefficients of variation provided in parentheses).

Filament	Datasheet			Experimental		
	Strain at Rupture %	Tensile Modulus MPa	Tensile Stress at Rupture MPa	Strain at Rupture % (CoV%)	Tensile Modulus MPa (CoV%)	Tensile Stress at Rupture MPa (CoV%)
Clear PLA ^a^	5.2	2346	45.6	2.0 (2)	1502 (5)	28.6 (10)
White PLA ^b^	6.0	3600	53.0	3.3 (18)	1578 (4)	46.9 (11)
PET-G ^c^	22.7	2020	50.4	6.1 (15)	872 (1)	36.3 (11)

^a^ Ultimaker, Technical data sheet PLA; ^b^ Filoalfa, Technical data sheet PLA; ^c^ RS, Product data sheet, PET-G.

#### Appendix A.1.3. Pull-Out Tests on Tubular Samples

In the pull-out tests, 50 mm long PLA and PETG tubular specimens were cast into mortar samples (75 mm × 75 mm × 255 mm) with embedment depths of 5 or 10 mm (Figure A2a). The mix constituents and proportions of the mortar comprised: CEM II/A-L1 32,5R cement (562 kg/m^3^); 0–2mm dried fine sand (562 kg/m^3^) and water (253 kg/m^3^). Once cured, the tubular specimens were pulled out of the mortar specimens at a rate of 0.05mm/s, using a servo-hydraulic universal machine with a load cell capacity of 100 kN, and observations made regarding their modes of failure.

The results are presented in terms of typical load-displacement responses in Figure A2b and pull-out or rupture loads in Table A2, noting the displacement is measured at the grips relative to the top of the mortar block.

**Table A2:** Pull-out test results.

		Sample	Material	Rib Pattern (mm)	Outer Diameter (mm)	Wall Thickness (mm)	Peak Force (N)	Bond Strength (MPa)	Rupture Stress (MPa)
Embedment Depth	10 mm	s_1	white PLA	annular pitch, 5	4	0.25	71.7	0.57	24.3
		s_2	PETG	annular pitch, 5	4	0.40	43.6	0.35	9.6
		s_3	clear PLA	spiral pitch,3	5	0.25	71.1	0.45	19.1
		s_4	clear PLA	spiral pitch,3	6	0.25	124.8	0.66	27.6
	5 mm	s_5	clear PLA	spiral pitch,3	5	0.25	93.0	1.18	24.9
		s_6	PETG	annular pitch, 5	4	0.40	81.2	1.31	14.2

#### Appendix A.1.4. Three-Point Bend Tests

The three-point bending tests were performed on notched mortar prisms (mix as in previous test) with embedded ink-filled tubes, in accordance with BS EN 12390-5. The details of tubes are given in Table A3, as illustrated in Figure A3.

In these tests, the load was controlled using feedback from a Crack Mouth Opening Displacement (CMOD) clip gauge, which was mounted on steel knife edges either side of the notch. The CMOD rate was set to 0.001 mm/s until a clear peak load had been reached, after which the rate of displacement change was gradually increased to a maximum rate of 0.02 mm/s. In addition, each test was paused for 180s three times during each test (i.e., at CMOD values of 0.1, 0.15 and 0.2 mm), to allow visual checks to be undertaken to determine if ink was visible on the surfaces of the beam. Results from these tests are given in Figure A3c.

**Table A3:** Three-point bend test results of mortar samples containing tubes.

Sample	Material	Rib Pattern (mm)	Outer Diameter (mm)	Wall Thickness (mm)	Peak Load (kN)
Control	-	-	-	-	2.9
s_1	clear PLA	spiral, pitch 5mm	5	0.25	2.2
s_2	clear PLA	annular, pitch 5mm	4	0.40	3.0
s_3	clear PLA	spiral, pitch 5mm	5	0.40	2.8
s_4	clear PLA	spiral, pitch 3mm	5	0.25	2.6

#### Appendix A.1.5. Conclusions from Preliminary Tests

The findings and conclusions from this initial set of tests are as followsIn relation to the direct tension tests carried out on solid bar specimens, the measured tensile strengths of all materials were lower than the values quoted in the material datasheets whilst the measured elongations at rupture were greater than the corresponding data-sheet values. These differences are attributed to the nature of 3D printed materials that are formed in imperfectly bonded layers.

The failure mode for all materials involved delamination of the printed layers, followed by complete rupture, which implies that local fractures form before final failure.

Applying the material data from the present direct tension bar tests to criterion 1 (Equation (A1)), suggests that tubular clear-PLA and white-PLA ligaments with spiral ribs (3 mm and 5 mm pitch) would rupture before the selected CMOD value (or very close to it), whilst the criterion would not be satisfied for the other material or ligament designs considered.

With the pull-out tests on tubular samples, all of the tubes ruptured, which suggests that the ribbed patterns considered provided adequate anchorage, even with the smaller embedment depths.

The stress at which the tubes ruptured were all lower than the corresponding material values from the solid bar tests. The two contributory factors to this trend are believed to be (i) bond weaknesses between 3D printed layers in the thin-walled tubes and (ii) stress concentrations adjacent to the ribs, both of which would have a proportionally larger effect than in the solid bars.

If it is assumed that the tubes are elastic up to first rupture, condition 1 (Equation (A1)) would suggest that all tubes would rupture before a crack opening reaches 0.1mm.

In relation to the three-point bend test, embedded tubes had a relatively small influence on the load-CMOD responses.

Only in sample 1 (clear PLA 5 mm tube with 0.25 mm wall thickness and 5mm spiral pattern) was ink visible at a CMOD value of 0.15 mm (which would equate to an opening of approximately 0.12 mm at the level of the tubes) although ink was visible at the end of all of the tests.

The average crack plane ink coverage, at the end of each test, was 70%, although this has a high variability and is difficult to determine accurately.

Both PLA materials are suitable for the MVNs although clear PLA tubes are more likely to rupture at the required serviceability crack opening.

Spiral rib patterns provide better bond than annular rib patterns and therefore are more likely to result in tube rupture at the required crack opening, with both 3 mm and 5 mm pitch rib patterns being viable, although 5mm are preferred from a 3D-printing viewpoint.

Rupture at the required crack opening is more likely to occur in thinner walled tubes (0.25 mm), although these have a greater permeability than thicker walled tubes (0.5 mm).

These conclusions informed the choice of MVN ligaments used in the main test series.

### Appendix A.2. Healing Agent

In order to establish the best prophylactic agent to be stored into the MVNs, the encapsulation environment and MVN sealing techniques, a number of viability tests were performed on cylindrical tubes (inner diameter = 2.5 mm, length 30 mm). Samples were filled with three different CA agents: (a) Procure PC20, of low viscosity (5 cPs¹); (b) Procure PC60, of medium viscosity (80–120 cPs¹); (c) Procure PC90, of low to medium viscosity (34–44 cPs¹). Samples were then sealed with silicon and broken at 24h intervals to determine the condition of the CA. CA is known to cure in the presence of moisture and therefore, to minimize the contact of the CA with moisture sources (from the external environment as well as from within the PLA capsule itself), five types of surface coatings were produced and tested. Samples were also placed in the desiccator (with silica gel RH = 30%, temp 20 °C ± 5 °C) for 24 h before being filled. The results of CA longevity tests are summarised in Table A4, where the state of CA is classified as liquid (l); partially solid with a liquid centre and solid annulus (s/l); and solid in all tubes (s).

**Table A4:** Longevity test of cyanoacrylate adhesives in clear PLA macro-capsules.

	Types of Coatings							Desiccator	State of CA		
	Waterproof PVA/Water 0.5:1		Waterproof PVA/Water 1:1		PVA Glue	Epoxy Hardener	Paraffin Wax	24 h	24 h	48 h	72 h
	1 Layer	2 Layers	1 Layer	2 Layers	1 Layer	1 Layer	1 Layer				
PC20									S ^a^		
	+							+	s		
		+						+	L ^b^	s/l ^c^	s
			+					+	l	s/l	s
				+				+	l	l	s
					+			+	s		
						+		+	l	s	
							+	+	l	s/l	s
PC60									l	s/l	s/l
							+	+	l	s/l	s/l
PC90									l	s	

^a^ solid in all tubes; ^b^ liquid in all tubes; ^c^ some solid, some with liquid centres with a solid annulus.
